# circRNA TCFL5 Promote Esophageal Cancer Progression by Modulating M2 Macrophage Polarization via the miR-543-FMNL2 Axis

**DOI:** 10.1155/2022/5075615

**Published:** 2022-05-18

**Authors:** Chengbin Lin, Yong Xi, Hongyan Yu, Zheng Wang, Xiaohan Chen, Weiyu Shen

**Affiliations:** Department of Thoracic Surgery, Ningbo Medical Center Lihuili Hospital, Ningbo University, Ningbo, Zhejiang 315040, China

## Abstract

**Objective:**

The mechanism of circRNA on M2 macrophage polarization, which contributes to esophageal cancer, remains unclear. This study is aimed at clarifying the mechanism of circRNA on esophageal cancer by regulating M2 macrophage polarization.

**Methods:**

The expression of circRNA TCFL5 and miR-543 was detected by qRT-PCR. Western blot was used to detect the expression of FMNL2 and CD163. CCK-8 and transwell assay was used to detect the proliferation, migration, and invasion of Eca109 and KYSE150, respectively. Flow cytometry was used to detect the CD163 positive cells. The contents of IL-10, TGF-*β*, TNF-*α*, IL-6, and IL-1*β* were detected by ELISA. A dual-luciferase reporter system was used to detect the regulation of miR-543 to circRNA TCFL5 and FMNL2.

**Results:**

156 upregulated circRNAs and 91 downregulated circRNAs in esophageal cancer tissues were identified, and the expression of circRNA TCFL5 showed the most significant upregulation. Overexpression of circRNA TCFL5 promotes proliferation, invasion, and migration of Eca109 and KYSE150 and promotes tumor growth *in vivo*. circRNA TCFL5 served as a sponge of miR-543, and FMNL2 was a downstream target gene of miR-543. circRNA TCFL5 promotes cell proliferation, migration, and invasion of Eca109 and KYSE150 by modulating the miR-543/FMNL2 axis. Macrophage M2 polarization promoted proliferation, invasion, and migration of Eca109 and KYSE150 cells, and circRNA TCFL5 mediated macrophage M2 polarization by regulating the FMNL2/miR-543 axis.

**Conclusion:**

In the present study, we identified that circRNA TCFL5 was dramatically upregulated in esophageal cancer, and circRNA TCFL5 promotes esophageal cancer progression by modulating M2 macrophage polarization via the miR-543-FMNL2 axis, which provides a potential target for the treatment of esophageal cancer.

## 1. Introduction

Esophageal cancer is one of the common malignant tumors in the digestive system. Its incidence and mortality rank eighth and sixth among malignant tumors, respectively. Esophageal squamous cell carcinoma (ESCC) is its primary pathological type [1, 2]. In recent years, although significant progress has been made in treating esophageal cancer, such as surgery, radiotherapy, and chemotherapy, the mortality remains high owing to its insidious onset and the advanced stage when they are diagnosed [3]. With molecular biology and precision medicine research, molecular targeted therapy has become an important research direction in tumor therapy [4]. It could provide new ideas for the prevention and treatment of esophageal cancer by exploring the specific mechanisms of the occurrence and development of esophageal cancer.

The immune microenvironment of the tumor and the various immune cells infiltrated in it are involved in many aspects of tumorigenesis and evolution, such as angiogenesis, immunosuppression, invasion, and metastasis [5]. Among these infiltrating immune cells, tumor-associated macrophages (TAMs) account for a higher proportion and are also the most important subgroup [6]. Clinical and experimental evidence indicated that macrophages infiltrating tumor tissue in most tumors generally have an M2-like phenotype [7, 8]. Increasing numbers of studies have found that TAMs are significantly related to the progression of esophageal cancer [9, 10]. Wang et al. found that FOXO1 promoted the polarization of M0 to M2 and the recruitment of M2 macrophages in the tumor microenvironment through the transcriptional regulation of CCL20 and CSF-1 to promote tumor proliferation [11]; Mai et al. revealed that compared with normal tissues, the expression of IL-33 in ESCC tissue was significantly increased, and the infiltration of M2-like macrophages into ESCC tumor tissue was significantly enhanced. Further studies indicated that IL-33 induces polarization of M2-like macrophages by activating ornithine decarboxylase, which helps form an immunosuppressive ESCC tumor microenvironment and promotes tumor progression [12]. Thus, we aimed to detect whether the M2 macrophages and immune responses are affected by the circular RNA (circRNA) we explored.

circRNA is a new member of the noncoding tumor genome, which is stable, conservative, universal, and specific. With the emergence of high-throughput sequencing technology and the development of bioinformatics, circRNA was found in different tissues. It can play an essential role in the proliferation, invasion, and metastasis of tumor cells through various mechanisms and esophageal cancer [13]. We found that the expression of circRNA TCFL5 was significantly increased in esophageal cancer according to circRNA sequencing results, indicating that circRNA TCFL5 might promote esophageal cancer progression. Recently, Lu et al. indicated that exosomal hsa-circ-0048117 promoted the polarization of M2 macrophages and promote the malignant behavior of ESCC cells, indicating that the circRNA may play a key role in reshaping the microenvironment and regulating the progress of ESCC [14]. However, how circRNA TCFL5 affects the progression of esophageal cancer and whether it controls M2 macrophages remain unknown. Therefore, this study intends to explore the function of circRNA TCFL5 on esophageal cancer progression and whether M2 macrophages are involved.

## 2. Materials and Methods

### 2.1. Patients and Samples

Thirty ESCC samples, cancer adjacent tissues, and serum samples were collected from 30 ESCC patients at Ningbo Medical Center Lihuili Hospital, Ningbo University, and were immediately frozen in liquid nitrogen and stored at -80°C. ESCC was confirmed by histopathological detection by two experienced pathologists. The Ningbo Medical Center Lihuili Hospital, Ningbo University Ethics Committee, approved this study, and all the patients have signed informed consent.

### 2.2. RNA Sequencing

Total RNA was extracted from three paired esophageal cancer samples and adjacent tissues. Nanodrop detected the RNA concentration, and the purity was analyzed using Bioanalyzer 2200 instrument (Aligent). The ribosomal RNA was removed by the RiboMinus Eukaryote Kit (Qiagen, Valencia, CA), and a cDNA library was constructed. circRNA sequencing was performed with Illumina HiSeq 3000 (Illumina). The differentially expressed circRNAs were screened with fold change > 2, and a *P* value < 0.05 was identified as an upregulated circRNA and as a downregulated lncRNA with the fold change < 0.5 with a *P* value < 0.05.

### 2.3. Cell Lines, Culture, and Treatment

The human esophageal cancer cell line Eca109 and KYSE150 cells were purchased from Hunan Fenghui Biotechnology Co., Ltd (CL0092; CL0493, Changsha, Hunan, China) and cultured in Dulbecco's modified Eagle's medium (DMEM; Gibco) supplemented with 10% fetal bovine serum (FBS, Gibco) at 37°C in a 5% CO_2_ atmosphere. The over-circRNA TCFL5, sh-circRNA TCFL5, miR-543 mimics, miR-543 inhibitor, over-FMNL2, and sh-FMNL2 were provided by GenePharma (Shanghai, China). Transfections of 50 nM miR-543 mimics, 100 nM miR-543 inhibitor, 50 nM sh-RNA, and 2 *μ*g RNA overexpressing vector were performed by using the Lipofectamine 3000 transfection reagent (Invitrogen) following the manufacturer's instruction when the confluence reached 60-90%. The function of these factors on cell proliferation, migration, and invasion was analyzed at 48 h after transfection.

### 2.4. RNA Extraction and qRT-PCR

The total RNA was extracted from the tissues and cells by TRIzol (Invitrogen). Subsequently, the RNA was reverse transcribed into cDNA using the RevertAid First Strand cDNA Synthesis Kit (Thermo). qRT-PCR was performed on an Applied Biosystems 7500 Real-Time PCR System (Applied Biosystems) by the SYBR Premix Ex Taq II Kit (Takara). GAPDH and U6 were used as the internal controls for mRNA and miRNA, respectively. The expression of circRNA TCFL5, miR-543, and FMNL2 was calculated by the 2^-*ΔΔ*Ct^ method. Primers for qPCR are as follows: TCFL5 F: 5′-TTTTGCGGTAAAACTGGCCG-3′, TCFL5 R: 5′-ACCTTCAGATTAATGACTGGCT-3′; circRNA TCFL5 F: 5′-ACATCAGACTAGACCTTTGTATGGG-3′, R: 5′-TGTTGCTGAAGAGGAACATTCA-3′; microRNA 543 F: 5′-ACTTAATGAGAAGTTGCCCGTG-3′, microRNA 543 R: 5′-GAAAAAGAAGTGCACCGCGA-3′; FMNL2 F: 5′-GTCCATGGGGTCAGAAGTGG-3′, FMNL2 R: 5′-TCACAGCTGCTAATCCTGAGT-3′; GAPDH F: 5′-GACAGTCAGCCGCATCTTCT-3′, GAPDH R: 5′-GCGCCCAATACGACCAAATC-3′; and U6 F: 5′-ATACAGAGAAAGTTAGCACGG-3′, U6 R: 5′-GGAATGCTTCAAAGAGTTGTG-3′.

### 2.5. Western Blot

The protein from cells or tissues was isolated by RIPA buffer, and the BCA method was used to detect the concentration. Subsequently, 30 *μ*g protein was separated by 8% SDS PAGE gel and transferred to the PVDF membrane. The membrane was incubated with primary antibody anti-FMNL2 (ab57963, 1 : 500, Abcam, Cambridge, UK), anti-CD163 (ab955, 1 : 1000, Abcam), and anti-GAPDH (ab8245, 1 : 500, Abcam) at 4°C with gentle shaking, overnight. After blocking with 5% nonfat dry milk (*w*/*v*) for 1 h. And then, the membrane was incubated with goat anti-mouse (ab6789, 1 : 2000, Abcam, Cambridge, UK) for 1 h at room temperature. Target proteins were visualized in a protein imaging system (Bio-Rad) with an enhanced chemiluminescence reagent.

### 2.6. Cell Counting Kit-8 (CCK-8) Assay

The Eca109 and KYSE150 cells were seeded into 96-well plates with a density of 4 × 10^3^ cells and then cultured for 12 h, 24 h, and 48 h in a CO_2_ incubator at 37°C. Subsequently, 10 *μ*L CCK-8 solution (Dojindo) was added into the cells in each well and incubated for 2.5 h at 37°C. A microplate reader detected the optical density (OD) value at 450 nm.

### 2.7. Cell Invasion and Migration

Cell invasion and migration ability were detected by the transwell assay with 8 *μ*m transwell inserts (Corning, USA). For the invasion assay, 50 *μ*L matrigel was used to coat the membranes, and 5 × 10^4^ Eca109 or KYSE150 cells in 200 *μ*L serum-free medium were added to the upper chamber. 600 *μ*L medium containing 20% FBS was added to the lower chamber. For the migration assay, the same procedures were needed except for the matrigel coating. Cells nonmigrating or noninvading in the upper chamber were removed after culturing for 24 h, and cells were fixed with 4% paraformaldehyde and stained with 0.1% crystal violet for 30 min. Moreover, the image was taken by a microscope.

### 2.8. Flow Cytometry

5 × 10^5^ macrophages with different treatments were trypsinized and were washed with 100 *μ*L sterilized PBS. Then, the cells were resuspended in 100 *μ*L PBS containing 10% FBS. Subsequently, 1 *μ*g FITC-labeled CD163 antibody was added to each tube and incubated at 4°C for 30 min. Next, cells were obtained, and free antibody was removed using sterilized PBS and resuspended cells with 300 *μ*L PBS containing 10% FBS. Cells were performed by the Attune NxT flow cytometer (Thermo Fisher, USA) and analyzed using the FlowJo software.

### 2.9. M2 Macrophage Induction

Ficoll-Hypaque density gradient centrifugation (GE Healthcare, USA) was used to isolate human peripheral blood mononuclear cells (PBMCs) from blood samples of patients with ESCC. The Human CD14 Positive Selection Kit (Stem Cell Technology, Vancouver, Canada) was used to isolate CD14^+^ monocytes from PBMCs according to the manufacturer's instructions. The monocytes were induced to differentiate into adherent macrophages after 5-day culture with 100 ng/mL M-CSF (PeproTech, NJ, USA) in 6-well plates (NEST Biotechnology, Wuxi, China) at 37°C. M2 macrophage polarization was induced at 20 ng/mL of IL-4 (ProteinTech Group, Inc.) for 48 h.

### 2.10. Dual-Luciferase Reporter Assay

Wild-type and mutant binding sites of circRNA TCFL5 and FMNL2 of miR-543 were cloned into the pmirGLO vector. The vectors and miR-543 mimics were transfected into the 293T cells by Lipofectamine 3000 reagents (Invitrogen) according to the instructions. Cells were lysed, and the relative luciferase activity was measured by dual-luciferase reporter assay kits (Promega, USA) after 48 hours of incubation.

### 2.11. Animal Experiments

4-5-week-old male BALB/c nude mice were purchased from Vital River company (Beijing, China) and divided into 3 groups at random. Subcutaneous tumor growth assays (*n* = 6 per group) were performed. 2 × 10^6^ KYSE150 cells stably expressing sh-circRNA-TCFL5 and over-circRNA-TCFL5 were injected into the right flank of nude mice. Mice in the control group were injected with KYSE150 cells without any transfection. Tumor size was measured every 7 days, and tumor volume was calculated with the formula: volume = 0.5 × *D* (longest diameter) × d2 (diameter perpendicular to the longest diameter). The animal experiments were approved by the Institutional Animal Care and Use Committee of Ningbo Medical Center Lihuili Hospital, Ningbo University.

### 2.12. Immunohistochemistry

Tumor tissues from patients or xenograft tumors were embedded in paraffin and cut into 4 *μ*m sections. 0.01 M citrate buffer was used for antigen retrieval of the sections after being dewaxed and hydrated. Subsequently, the sections were incubated with 3% H_2_O_2_ for 15 min and goat serum for 30 min at room temperature, respectively. And then, the sections were incubated with primary antibodies anti-CD163 (1 : 50, sc-20066, Santa Cruz, CA, USA) and anti-Ki67 (1 : 50, sc-23900, Santa Cruz) overnight at 4°C, and then incubated with horseradish peroxidase- (HPR-) labeled secondary antibody (1 : 200, Boster) for 30 min at room temperature and stained with diaminobenzidine (DAB) and hematoxylin. The sections were observed under a microscope after being sealed in neutral resin.

### 2.13. TUNEL Assay

Proteinase K was used to treat tumor tissue for 30 min and incubation with 3% H_2_O_2_ and 0.1% TritonX-100 for 10 min at room temperature. And then, the TUNEL assay was performed according to the instructions (Roche, USA). Briefly, 10 *μ*L TUNEL solution was used to biotinylate sections and then stained with DAB and hematoxylin. The sections were observed under a microscope after being sealed in neutral resin.

### 2.14. ELISA

The contents of IL-10, TGF-*β*, TNF-*α*, IL-6, and IL-1*β* in serum samples of esophageal cancer patients were tested by the ELISA assay. In brief, cells with different treatments were collected, and then, the concentration of IL-10, TGF-*β*, TNF-*α*, IL-6, and IL-1*β* was determined using an ELISA kit obtained from R&D Systems (D1000B; DB100B; DTA00D; D6050; DLB50; Minneapolis, MN, USA) following the manufacturer's protocols.

### 2.15. Statistical Analysis

All the data in the present study were analyzed with Prism 6.0 (GraphPad Software, USA) and expressed as the mean ± standard deviation (SD). Student's *t*-test or one-way analysis of variance (ANOVA) followed by Tukey's post hoc test was used for statistical analysis between two groups or multiple comparisons. *P* < 0.05 was considered statistically significant.

## 3. Results

### 3.1. circRNA TCFL5 Was Identified as a Critical circRNA That Was Highly Expressed in Esophageal Cancer

**Figure 1:**
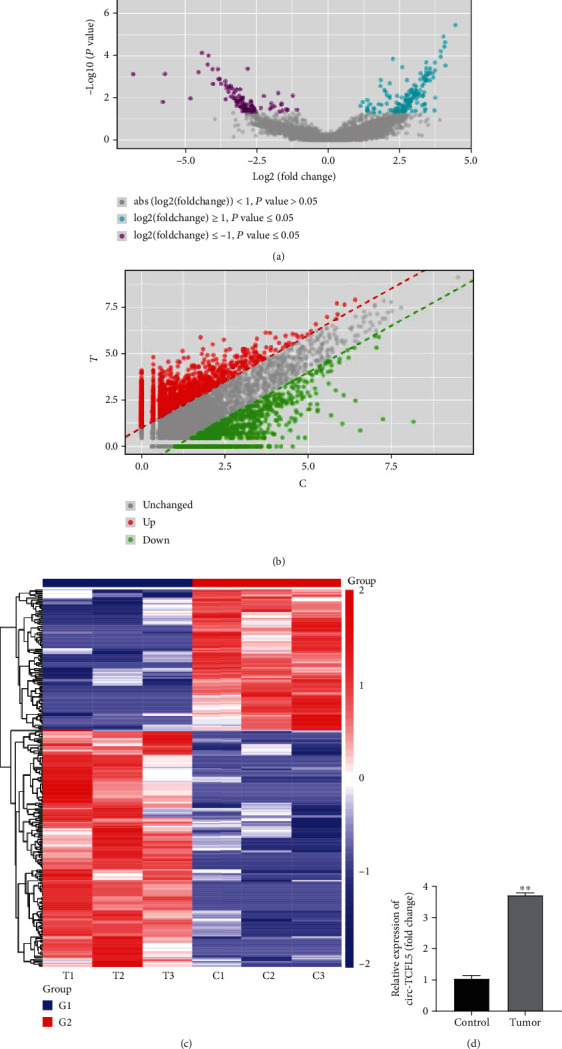
circRNA TCFL5 identified as a critical circRNA that was highly expressed in esophageal cancer. (a–c) The differentially expressed circRNAs in esophageal cancer tissues detected by circRNA sequencing were presented in the volcano, scatter, and heat maps, respectively; (d) qRT-PCR was used to detect the expression of circRNA TCFL5 in the esophageal cancer tissues. Data are shown as the mean ± SD. ^∗∗^*P* < 0.01, vs. control group.

To explore the circRNAs related to esophageal cancer, circRNA sequencing analysis on esophageal cancer tissues was performed. The circRNA sequencing results showed 247 differentially expressed circRNAs in esophageal cancer tissues compared with the control group, including 156 upregulated circRNAs and 91 downregulated circRNAs. The volcano, scatter, and heat maps presented the differentially expressed circRNAs in esophageal cancer tissues (Figures 1(a)–1(c)). Of them, the expression of circRNA TCFL5 showed the most upregulated in esophageal cancer tissues compared with the control group. qRT-PCR analysis showed that the expression of circRNA TCFL5 was dramatically upregulated in the tumor group compared with the control group (Figure 1(d)), suggesting that circRNA TCFL5 might promote esophageal cancer progression.

### 3.2. Overexpression of circRNA TCFL5 Promotes Proliferation, Invasion, and Migration of Eca109 and KYSE150

**Figure 2:**
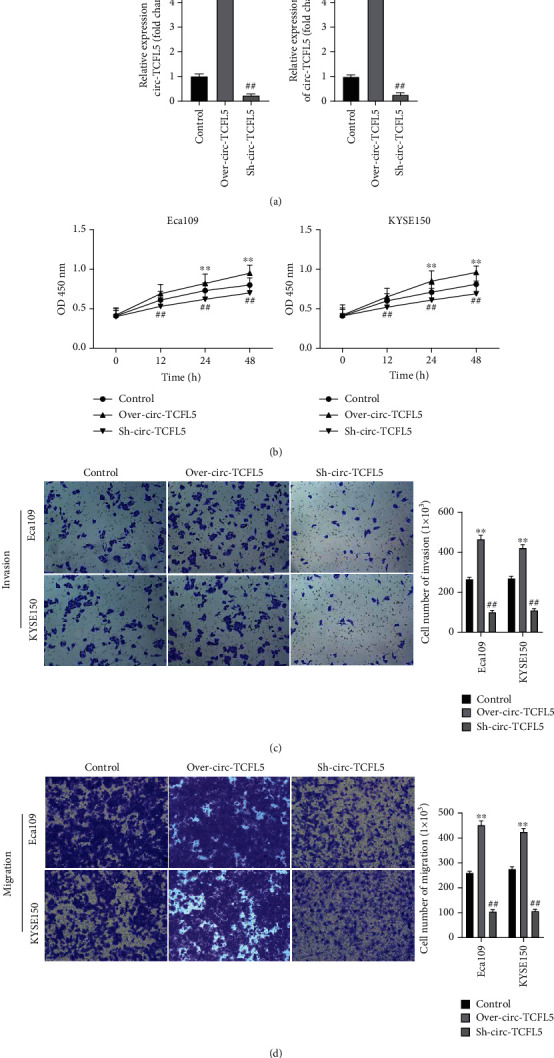
Overexpression of circRNA TCFL5 promotes proliferation, invasion, and migration of Eca109 and KYSE150. (a) qRT-PCR was used to detect whether the expression of circRNA TCFL5 is overexpressed or suppressed Eca109 and KYSE150 cells; (b) CCK-8 analysis was used to detect the proliferation of Eca109 and KYSE150 cells affected by circRNA TCFL5; (c, d) transwell assay was performed to detect the migration and invasion of Eca109 and KYSE150 cells affected by circRNA TCFL5. Data are shown as the mean ± SD. ^∗∗^*P* < 0.01, over-circRNA TCFL5 vs. NC group; ^##^*P* < 0.01, Sh-circRNA TCFL5 vs. NC group.

The function of circRNA TCFL5 on esophageal cancer progression was investigated. Overexpression and knockdown of circRNA TCFL5 Eca109 and KYSE150 cells were constructed. qRT-PCR confirmed that circRNA TCFL5 was successfully overexpressed or suppressed in Eca109 and KYSE150 cells (Figure 2(a)). CCK-8 analysis showed that proliferation was significantly increased when overexpressing circRNA TCFL5 while decreased when suppressing circRNA TCFL5 compared with the NC group (Figure 2(b)). The transwell assay indicated that overexpression of circRNA TCFL5 enhanced the migration and invasion of Eca109 and KYSE150 cells. On the contrary, knockdown of circRNA TCFL5 Eca109 decreased the migration and invasion abilities of the Eca109 and KYSE150 cells compared with the NC group (Figures 2(c) and 2(d)).

### 3.3. circRNA TCFL5 Served as a Sponge of miR-543

**Figure 3:**
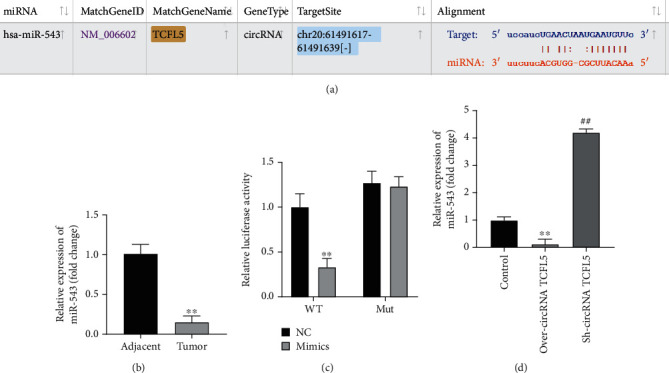
circRNA TCFL5 served as a sponge of miR-543. (a) Starbase 2.0 was used to predict the miRNA targets of circRNA TCFL5 and the potential target of circRNA TCFL5 and miR-543. (b) qRT-PCR analysis was used to detect miR-543 in esophageal cancer tissues, ^∗∗^*P* < 0.01, vs. control group. (c) Luciferase reporter assays were used to detect the binding between miR-543 and circRNA TCFL5 in Eca109 cells, *P* < 0.01, vs. NC group. (d) The expression of miR-543 affected by circRNA TCFL5 in Eca109 cells was detected by qRT-PCR. Data are shown as the mean ± SD, ^∗∗^*P* < 0.01, over-circRNA TCFL5 vs. NC group; ^##^*P* < 0.01, Sh-circRNA TCFL5 vs. NC group.

It has been proved that circRNA could act as sponges of miRNA. In order to explore the sponge of circRNA TCFL5, Starbase 2.0 (https://starbase.sysu.edu.cn/starbase2/index.php) was used to predict the miRNA targets of circRNA TCFL5. We found that miR-543 was a potential target of circRNA TCFL5 (Figure 3(a)). qRT-PCR analysis showed that the expression of miR-543 was dramatically decreased in esophageal cancer tissues compared with normal tissues (Figure 3(b)). Luciferase reporter assays indicated that miR-543 mimic significantly inhibited the luciferase activity containing wild-type binding sites of circRNA TCFL5 but not affected by containing mutant binding sites of circRNA TCFL5 (Figure 3(c)). In addition, we found that the expression of miR-543 was significantly downregulated in the over-circRNA TCFL5 group while upregulated in the sh-circRNA TCFL5 group compared with the NC group (Figure 3(d)). These results indicated that circRNA TCFL5 served as a sponge of miR-543.

### 3.4. FMNL2 Was a Downstream Target Gene of miR-543

**Figure 4:**
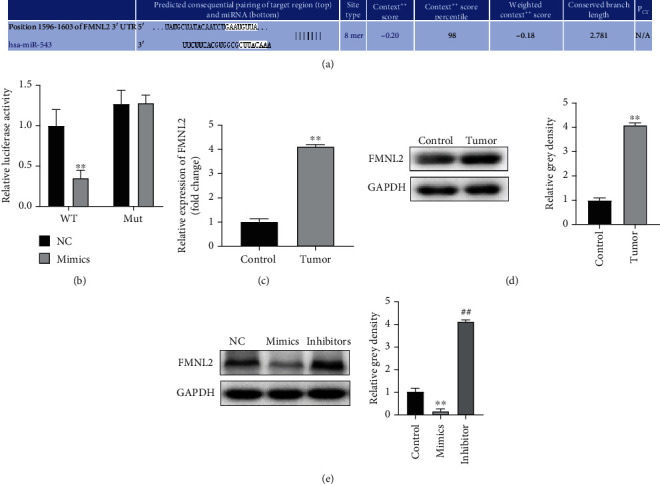
FMNL2 was a downstream target gene of miR-543. (a) TargetScan was used to predict the targets of miR-543 and a binding site on position 1596-1603 at the 3′UTR of FMNL2. (b) Dual-luciferase reporter gene assay was used to detect the binding between miR-543 and FMNL2 in Eca109 cells. ^∗∗^*P* < 0.01, vs. NC group. (c, d) qPCR and western blot analysis were used to detect the expression of FMNL2 in esophageal cancer tissues, *P* < 0.01 vs. control group. (e) The expression of FMNL2 in Eca109 cells affected by miR-543 was detected by western blot. Data are shown as the mean ± SD, ^∗∗^*P* < 0.01, miR-543 mimics vs. NC group; ^##^*P* < 0.01, miR-543 inhibitor vs. NC group.

In order to know the target gene of miR-543, TargetScan (https://www.targetscan.org/vert_71/) was used to predict the targets of miR-543. We found that FMNL2 was one of the targets of FMNL2 that has a binding site on position 1596-1603 at the 3′UTR of FMNL2 (Figure 4(a)). The dual-luciferase reporter gene assay indicated that miR-543 mimics significantly reduced the luciferase activity by cotransfection with wild-type binding sites of FMNL2 while luciferase activity was not affected by cotransfection with mutant binding sites of FMNL2 (Figure 4(b)). Furthermore, qPCR and western blot analysis showed that the expression of FMNL2 was dramatically increased in esophageal cancer tissues compared with normal tissues (Figures 4(c) and 4(d)). In addition, we found that miR-543 mimics could decrease the expression of FMNL2 while miR-543 inhibitor increased FMNL2 expression (Figure 4(e)), suggesting that FMNL2 is a direct target of miR-543.

### 3.5. circRNA TCFL5 Promote Cell Proliferation, Migration, and Invasion of Eca109 and KYSE150 by Modulating the miR-543/FMNL2 Axis

**Figure 5:**
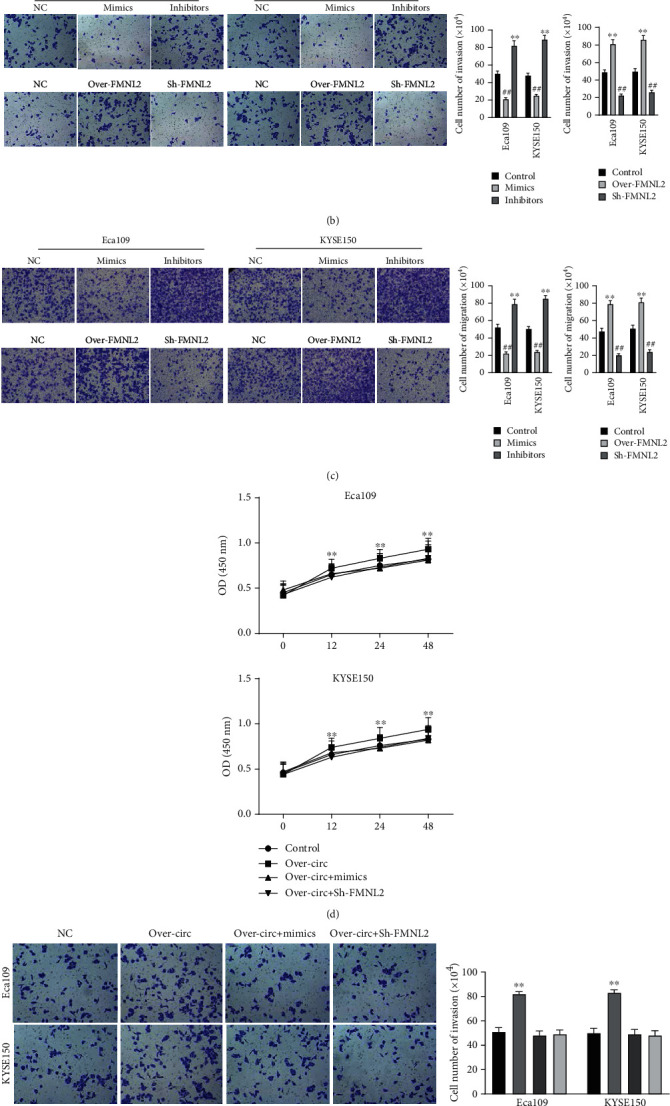
circRNA TCFL5 promotes cell proliferation, migration, and invasion of Eca109 and KYSE150 by modulating the miR-543/FMNL2 axis. (a) CCK-8 analysis was used to detect the proliferation of Eca109 and KYSE150 cells affected by miR-543 or FMNL2; (b, c) transwell assay was performed to detect the migration and invasion of Eca109 and KYSE150 cells affected by miR-543 or FMNL2; ^∗∗^*P* < 0.01, miR-543 inhibitors, or over-FMNL2 groups vs. NC group; ^##^*P* < 0.01, miR-543 mimics or Sh-FMNL2 groups vs. NC group; (d) CCK-8 analysis was used to detect the proliferation of Eca109 and KYSE150 cells affected by circRNA TCFL5 and reversed by miR-543 or FMNL2; (e, f) transwell assay was performed to detect the migration and invasion of Eca109 and KYSE150 cells affected by circRNA TCFL5 and reversed by miR-543 or FMNL2. Data are shown as the mean ± SD. ^∗∗^*P* < 0.01, over-circRNA TCFL5 vs. NC group.

CCK-8 and transwell assay indicated that miR-543 mimics and downregulation of FMNL2 could inhibit cell proliferation, migration, and invasion compared with the control group. On the contrary, the miR-543 inhibitor group and overexpression of FMNL2 promoted cell proliferation, migration, and invasion compared with the control group (Figures 5(a)–5(c)). In addition, we found that miR-543 mimics or suppression of FMNL2 could partially rescue the function of circRNA TCFL5 overexpression on cell proliferation, migration, and invasion (Figures 5(d)–5(f)), suggesting that hsa_circ_0061129 circRNA TCFL5 promote cell proliferation, migration, and invasion of Eca109 and KYSE150 by modulating the miR-543/FMNL2 axis.

### 3.6. Overexpression of circRNA TCFL5 Promoted Esophageal Cancer Progression In Vivo

**Figure 6:**
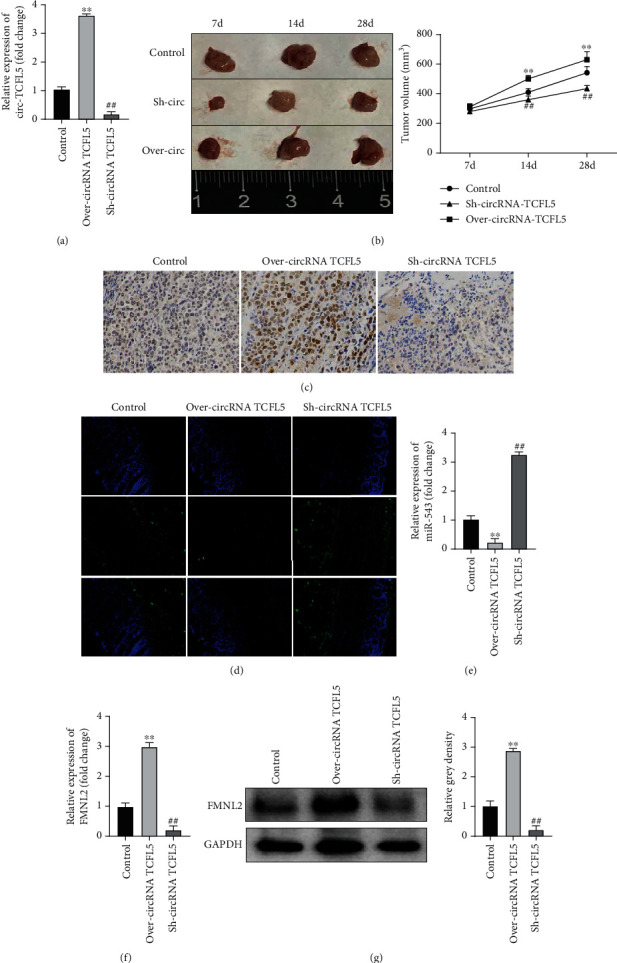
Overexpression of circRNA TCFL5 promoted esophageal cancer progression *in vivo*. (a) The expression of circRNA TCFL5 was successfully upregulated or suppressed in xenograft tumors. (b) The tumor volume was remarkably higher with circRNA TCFL5 overexpression while lower with circRNA TCFL5 suppression than that in the NC group. (c) Ki-67 immunohistochemistry was used to detect the proliferation affected by circRNA TCFL5. (d) TUNEL analysis was used to detect the tumor cells with apoptosis affected by circRNA TCFL5. (e) qPCR analysis was used to detect the expression of miR-543 affected by circRNA TCFL5. (f, g) The expression of FMNL2 was detected by qRT-PCR and western blot affected by circRNA TCFL5, respectively. Data are shown as mean ± SD, ^∗∗^*P* < 0.01, over-circRNA TCFL5 vs. NC group; ^##^*P* < 0.01, Sh-circRNA TCFL5 vs. NC group.

Xenograft tumors in nude mice were established to confirm the function of circRNA TCFL5 promoting esophageal cancer progression *in vivo*. As shown in Figure 6(a), the expression of circRNA TCFL5 was successfully upregulated or suppressed in xenograft tumors. Further analysis indicated that the tumor volume was remarkably higher with circRNA TCFL5 overexpression and lower with circRNA TCFL5 suppression than in the NC group (Figure 6(b)). Ki-67 immunohistochemistry revealed that the positive Ki-67 cells were significantly increased when overexpressing circRNA TCFL5 while decreased when suppressing circRNA TCFL5 than the NC group (Figure 6(c)). TUNEL analysis indicated that the apoptosis was significantly decreased in the overexpressing circRNA TCFL5 group while dramatically increased in the suppressing circRNA TCFL5 group compared with the NC group (Figure 6(d)). In addition, the expression of miR-543 and FMNL2 was detected in the xenograft tumors. qPCR analysis showed that the expression of miR-543 was significantly downregulated in the circRNA TCFL5 group while elevated in the circRNA TCFL5 group (Figure 6(e)). The expression of FMNL2 showed the opposite results compared with the expression changes of miR-543 affected by circRNA TCFL5 (Figures 6(f) and 6(g)). These results demonstrated that overexpression of circRNA TCFL5 promoted esophageal cancer progression by regulating the miR-543/FMNL2 axis *in vivo*.

### 3.7. Macrophage M2 Polarization Promoted Proliferation, Invasion, and Migration of Eca109 and KYSE150 Cells

**Figure 7:**
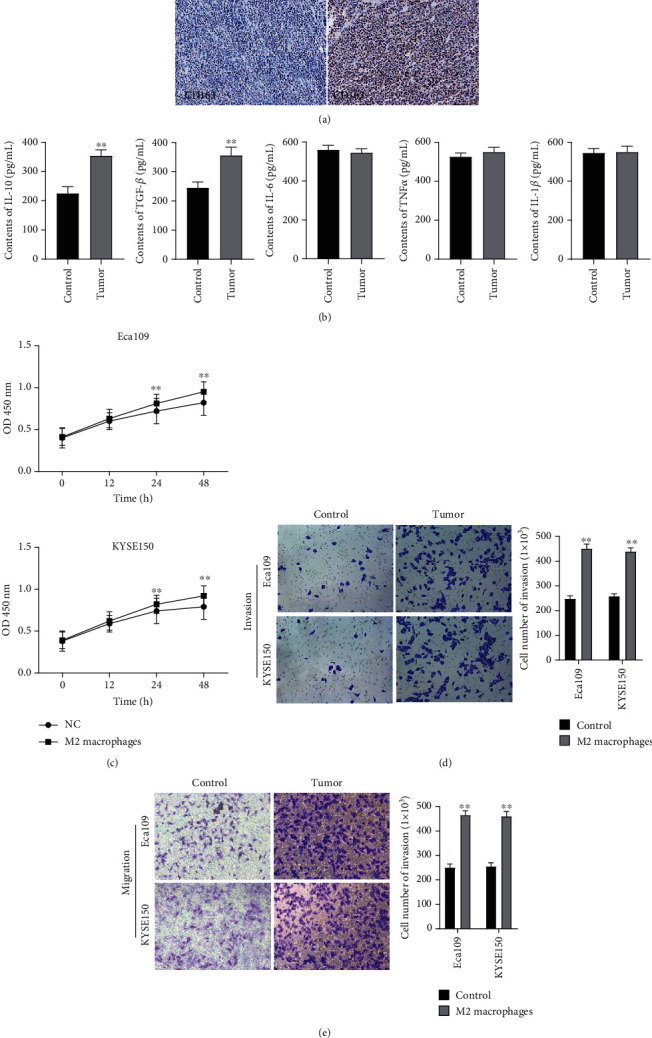
Macrophage M2 polarization promoted proliferation, invasion, and migration of Eca109 and KYSE150 cells. (a) The expression of CD163 was detected by immunohistochemistry in esophageal cancer tissues; (b) ELISA was used to detect the contents of IL-10, TGF-*β*, TNF-*α*, IL-6, and IL-1*β* in esophageal cancer tissues; ^∗∗^*P* < 0.01 vs. NC group; (c) CCK-8 analysis was used to detect the proliferation of Eca109 and KYSE150 cells affected by macrophage M2 polarization; (d, e) transwell assay was performed to detect the migration and invasion of Eca109 and KYSE150 cells affected by macrophage M2 polarization. Data are shown as mean ± SD, ^∗∗^*P* < 0.01, vs. NC group.

To explore the role of M2 macrophages in the inflammatory responses related to esophageal cancer, we first verified the content of M2 macrophages in esophageal cancer. The results showed that compared with healthy controls, the expression of CD163, a marker of M2 macrophages, was significantly increased in esophageal cancer tissues (Figure 7(a)). Further analysis showed that the contents of IL-10 and TGF-*β* was significantly increased in tumor tissues, but there were no significant changes of TNF-*α*, IL-6, and IL-1*β* observed compared with the control group (Figure 7(b)). And then, the function of M2 macrophages on Eca109 and KYSE150 was detected by coculturing with M2 macrophages and Eca109 and KYSE150. CCK-8 analysis indicated that M2 macrophages accelerated the proliferation of Eca109 and KYSE150 cells compared with the control group (Figure 7(c)). The transwell assay indicated that M2 macrophages dramatically promoted the invasion and migration of Eca109 and KYSE150 compared with the control group (Figures 7(d) and 7(e)). The results indicated that M2 macrophages play an essential role in promoting the occurrence and development of esophageal cancer.

### 3.8. circRNA TCFL5 Mediated Macrophage M2 Polarization by Regulating FMNL2/miR-543 Axis

**Figure 8:**
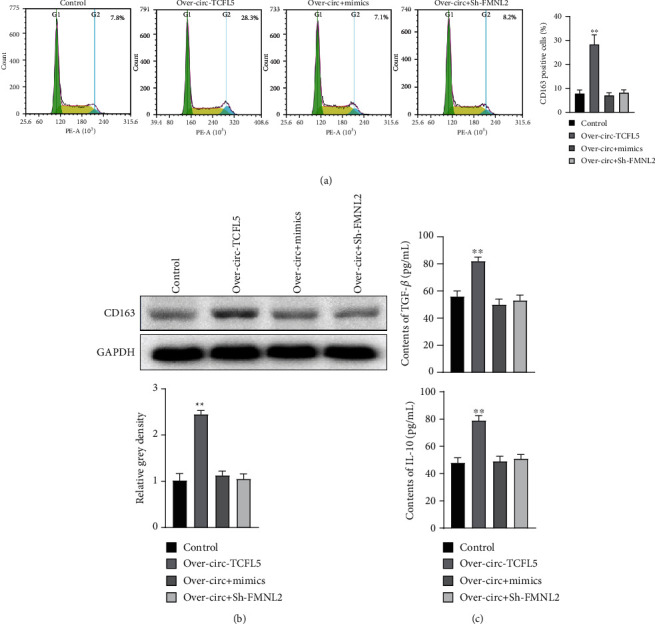
circRNA TCFL5 mediated macrophage M2 polarization by regulating FMNL2/miR-543 axis. (a) Flow cytometric assay was used to detect the CD163 positive cells affected by over-circRNA TCFL5. (b) The expression of CD163 was detected by western blot affected by circRNA TCFL5 and reversed by miR-543 mimics or suppression of FMNL2. (c) The contents of IL-10 and TGF-*β* were detected by ELISA affected by circRNA TCFL5 and reversed by miR-543 mimics or suppression of FMNL2. Data are shown as mean ± SD, ^∗∗^*P* < 0.01, over-circRNA TCFL5 group vs. NC group.

In order to know the function of circRNA TCFL5 on macrophage M2 polarization, circRNA TCFL5 was overexpressed in the over-circRNA TCFL5 group and then was induced to M2 polarization. The flow cytometric assay indicated that the CD163 positive cells were significantly increased in the over-circRNA TCFL5 group compared with the NC group (Figure 8(a)). Further analysis showed that the expression of CD163 was significantly upregulated when overexpressing circRNA TCFL5 compared with the NC group (Figure 8(b)). Furthermore, the contents of IL-10 and TGF-*β* were significantly increased when overexpressing circRNA TCFL5 compared with the NC group (Figure 8(c)). In addition, we found that miR-543 mimics or suppression of FMNL2 could partially rescue the function of circRNA TCFL5 overexpression on macrophage M2 polarization (Figures 8(a)–8(c)). These results indicated that circRNA TCFL5 mediated macrophage M2 polarization by regulating the FMNL2/miR-543 axis.

## 4. Discussion

circRNA has evolutionary conservation in different species and usually has expression specificity in different tissues and developmental stages [15, 16]. Multiple circRNAs have been confirmed to play a vital role in the occurrence and development of tumors and can be used as new tumor markers [17]. Similarly, studies have found that circRNAs can play an essential role in esophageal cancer by regulating different biological processes [18]. For example, Hsa_circ_0006948 affects mesenchymal transformation and promotes the development of esophageal cancer by regulating the miR-490-3p/HMGA2 signal axis [19]. circRNA LPAR3 can promote the development of liver cancer by promoting the invasion, migration, and metastasis of esophageal cancer cells [20]. In the present study, 156 upregulated circRNAs and 91 downregulated circRNAs were identified, and circRNA TCFL5 showed the most upregulated in esophageal cancer tissues compared with the control group. Further analysis indicated that overexpressing circRNA TCFL5 promoted esophageal cancer progression both in vitro and *in vivo*. It is the first report that clarifies the function of circRNA TCFL5 in esophageal cancer progression.

Increasing numbers of studies have proved that circRNA could participate in esophageal cancer development by sponging miRNAs that regulate the target gene's expression. Liu et al. demonstrated that circRNA_100367 promoted the radiation sensitivity of esophageal squamous cell carcinomas by downregulation of Wnt3 via sponging miR-217 [21]. circRNA_001275 promotes cisplatin resistance by upregulating Wnt7a expression via competitively sponging miR-370-3p in esophageal cancer [22]. Circular RNA LPAR3 sponges microRNA-198 to facilitate esophageal cancer migration, invasion, and metastasis [20]. In the present study, we found that circRNA TCFL5 served as a sponge of miR-543, and FMNL2 was a downstream target gene of miR-543. miR-543 has been demonstrated as a tumor suppressor in different cancers, including colorectal cancer, glioma, and breast cancer [23–26]. In our work, we indicated that miR-543 inhibits proliferation, migration, and invasion of esophageal cancer cells, which is consistent with previous reports. However, the function of miR-543 on esophageal cancer cells could be partially reversed by circRNA TCFL5. FMNL2 was also demonstrated to promote cancer malignant development, such as FMNL2 was considered a positive regulator of cell motility and metastasis in colorectal carcinoma [27]. Moreover, circHIPK3 promotes proliferation and metastasis of colorectal cancer cells via upregulation of FMNL2 by sponging miR-1207-5p [28]. In addition, FMNL2 was found upregulated in esophageal cancer by bioinformatic analysis of GSE6188, GSE13937, and GSE43732 microarrays [29], suggesting that FMNL2 might act as an oncogene in esophageal cancer. In the present study, we demonstrated that overexpressing FMNL2 promoting miR-543 could partially reverse esophageal cancer development. These results demonstrated that circRNA TCFL5 promoted esophageal cancer development by upregulating FMNL2 via sponging miR-543.

In recent years, with the in-depth study of the tumor structure, people have fully realized that the formation and evolution of tumor cells are related to cancer cells themselves and closely related to the microenvironment in which cancer cells live. TAMs are one of the most critical components in the microenvironment, which plays an essential role in the immune microenvironment of the tumor by regulating tumor angiogenesis, degrading the extracellular matrix, and secreting some immunosuppressive factors. Increasing numbers of evidence indicated that circRNAs are closely related to innate and adaptive immune responses and can regulate the differentiation and function of immune cells, such as T cells, dendritic cells (DC), and macrophages. Recently, Lu et al. indicated that exosomal hsa-circ-0048117 could promote the malignant behavior of ESCC cells by promoting the polarization of M2 macrophages [14], suggesting that circRNA participated in TAM regulation, which contributes to esophageal cancer progression. In the present study, we demonstrated that the polarization of M2 macrophages could promote esophageal cancer development. Further analysis revealed that circRNA TCFL5 promoted the polarization of M2 macrophages by regulating the FMNL2/miR-543 axis. Taken together, we demonstrated that circRNA TCFL5 promotes esophageal cancer progression by modulating M2 macrophage polarization via the miR-543-FMNL2 axis.

In conclusion, we identified that circRNA TCFL5 was dramatically upregulated in esophageal cancer and could promote esophageal cancer progression both in vitro and *in vivo*. Mechanically, we demonstrated that circRNA TCFL5 promotes esophageal cancer progression by modulating M2 macrophage polarization via the miR-543-FMNL2 axis. These findings provide a basis for targeted therapy of esophageal cancer that dramatically reduces esophageal cancer mortality and improves patients' quality of life.

## Data Availability

The data used to support the findings of this study are available from the corresponding authors upon request.
